# Home-based carer-assisted therapy for people with stroke: findings from a randomised controlled trial

**DOI:** 10.1186/1471-2458-12-S2-A2

**Published:** 2012-11-27

**Authors:** Nor Azlin Mohd Nordin, Noor Azah Aziz, Saperi Sulong, Syed Mohamed Aljunid

**Affiliations:** 1 Faculty of Health Sciences, Universiti Kebangsaan Malaysia, Jalan Raja Muda Abdul Aziz, 50300 Kuala Lumpur, Malaysia; 2 United Nations University-International Institute for Global Health, Universiti Kebangsaan Malaysia Medical Centre, Jalan Yaacob Latiff, 56000 Kuala Lumpur, Malaysia; 3 Department of Family Medicine, Universiti Kebangsaan Malaysia Medical Centre, Jalan Yaacob Latiff, 56000 Kuala Lumpur, Malaysia; 4 Department of Health Information, Universiti Kebangsaan Malaysia Medical Centre, Jalan Yaacob Latiff, 56000 Kuala Lumpur, Malaysia

## Background

The benefits of engaging carers in the rehabilitation of people with stroke have not been well-researched despite emphasis on shared responsibility between healthcare providers and the stroke patient’s family. This study aimed to assess the effectiveness of a task-oriented training assisted by carer for stroke patients living at home following hospital rehabilitation.

## Materials and methods

A single-blinded randomised controlled trial was conducted on 91 stroke patients. In all, 76.5% males with mean age of 58.9±10.6 years and median stroke duration of 13.0 months (range 6-84) completed intensive rehabilitation at a tertiary hospital. The control group received outpatient group exercise led by therapists while the experimental group was assigned to a home-based family-assisted task-oriented training. Primary outcomes were mobility (Rivermead Mobility Scale), balance (Berg’s Balance Scale), lower limb strength (5-Times-Sit-to-Stand Test) and gait speed. Secondary outcome was health-related quality of life as measured using EQ5D-Visual analogue Scale. All assessments were carried out at baseline and at week twelve of intervention. An intention-to-treat analysis was used to evaluate outcome of the interventions.

## Results

No statistical differences were found between the experimental and the control group in all outcomes (all p>0.25) at completion of the trial. Both groups improved significantly in all the measures of function; mobility (p<0.01), balance (p<0.01), lower limb strength (p<0.01), gait speed (p<0.05), and in the quality of life score (p<0.05). The study participants showed meaningful progress in gait speed (mean gain >8.0 m/min) after twelve weeks of intervention irrespective of the therapy group.

## Conclusion

The home-based carer-assisted therapy is as effective as the outpatient therapist-led training in improving post-stroke functions and quality of life. The programme may be considered as part of a discharge or long-term care plan for stroke patients following hospital rehabilitation.

